# Changes in social behavioral developmental risks in preschool children after the first COVID-19 wave: a prospective longitudinal cohort study

**DOI:** 10.1038/s41598-023-32877-x

**Published:** 2023-04-06

**Authors:** Anika Kästner, Vanessa Sophie Ernst, Wolfgang Hoffmann, Marco Franze

**Affiliations:** Institute for Community Medicine, Section Epidemiology of Health Care and Community Health, University Medicine Greifswald, Ellernholzstr. 1-2, 17487 Greifswald, Germany

**Keywords:** Epidemiology, Paediatric research, Population screening, Paediatric research

## Abstract

The impact of the COVID-19 pandemic on social-emotional developmental risks (SE-DR) of preschool children is largely unknown. Therefore, the aim of this prospective longitudinal dynamic cohort study was to assess changes in preschoolers’ SE-DR from before the pandemic to after the first COVID-19 wave. SE-DR were assessed annually with the instrument “Dortmund Developmental Screening for Preschools” (DESK). Longitudinal DESK data from 3- to 4-year-old children who participated both in survey wave (SW) three (DESK-SW3, 2019) and SW four (DESK-SW4, 2020) from August 1 to November 30 were used, respectively. Additionally, data from previous pre-pandemic SW were analyzed to contextualize the observed changes (SW1: 2017; SW2: 2018). A total of *N* = 786 children were included in the analysis. In the pre-pandemic DESK-SW3, the proportion of children with SE-DR was 18.2%, whereas in DESK-SW4 after the first COVID-19 wave, the proportion decreased to 12.4% (*p* = 0.001). Thus, the prevalence rate ratio (PRR) was 0.68. Compared to data from previous SW (SW1-SW2: PRR = 0.88; SW2-SW3: PRR = 0.82), this result represents a notable improvement. However, only short-term effects were described, and the study region had one of the highest preschool return rates in Germany. Further studies are needed to examine long-term effects of the pandemic on preschoolers’ SE-DR.

## Introduction

Since the outbreak of the COVID-19 pandemic in March 2020, the lives of many people have drastically changed, with children in particular being affected in their daily lives by preschool and school closures and limited childcare options. In Germany, emergency child care services were offered to preschool children of parents with system-relevant occupations (e.g. medical staff, police, staff providing emergency daycare in preschools and schools)^1^. For instance in the German federal state Mecklenburg Western-Pomerania, the percentage of preschools that provided such services ranged between 0–15% (March until April 2020) and 15–77% (May until June 2020), respectively^2^. Regular operation of preschools was not possible until August 2020.

Several studies were initiated in Europe with a focus on transmission of SARS-CoV-2 and the impact of hygiene measures among children aged 0 to 19 years^3,4^. However, little is known about the impact of the pandemic on developmental risks in preschool children. In particular, the promotion of social-emotional competencies in preschools is crucial for later school success, mental health and contributes to the reduction of social inequalities^5–8^. Therefore, the loss of pedagogical promotion of children’s competencies in preschools might especially increase the prevalence of developmental risks in the social-emotional domain.

When assessing the development of children's social-emotional competencies, two general distinctions of social-emotional problems are made: Externalizing problem behavior refers to directing emotional responses away from the self and is characterized by disruptive behavior (e.g. aggression and rule-breaking behavior), whereas internalizing problem behavior implies that emotional responses are directed inward and is associated with anxiety, depression and psychosomatic problems^9–11^. Generally, social-emotional functioning can be considered a basic competency contributing to mental health and well-being. With regard to the effects of the lockdown on social-emotional competencies of preschool children, studies found that internalizing as well as externalizing problems significantly increased^12^. Interestingly, the subgroup of preschoolers who met the World Health Organization recommendations for physical activity had lower internalizing scores than non-active peers^12^. Most parents and children experienced lockdown-related stress and especially single parenthood and being an only child were associated with higher levels of child problems, e.g., more emotional symptoms^13,14^. Children and adolescents were more likely to experience higher rates of depression and anxiety symptoms during and after enforced isolation relative to before the pandemic^15–18^. Browne et al. showed that the mental health of Canadian preschool- to school-aged boys in early childhood education significantly deteriorated after the onset of the pandemic^19^. One study by Specht et al. showed a modest decrease in child-emotional behavioral functioning during the COVID-19 lockdown, potentially due to parental stress^20^.

One longitudinal study from Japan examined the social-emotional behavior with the Strengths and Difficulties Questionnaire (SDQ) of 4–6-year-old preschoolers during the first COVID-19 wave compared to after preschool closure and found no significant differences in preschoolers’ socio-emotional behavior^21^. Shum et al. longitudinally examined psychosocial difficulties in 2–5-year-old preschool children from North West England starting mid-April to July 2020 and found no significant differences one month later^22^. Interestingly, in another study applying the same study setting as Shum et al., the authors found an increase in socio-emotional difficulties in primary school aged children, and mixed results in secondary school-aged children: an increase in restlessness/attention difficulties, a decrease in emotional difficulties, and no change in behavioral difficulties^23^. When comparing the SDQ in preschoolers before (January 2020) versus during restrictions (May 2020), a Finnish study with a very small sample size (n = 22) showed no longitudinal changes in the scales emotional and behavioral difficulties, hyperactivity, and peer relationship problems, however, prosocial skills were significantly reduced^24^.

Despite some lessons learned, most studies to date have not analyzed longitudinal data before the onset of the COVID-19 pandemic in preschool-aged children, or questionnaires were completed by parents only^12,13^. Most studies in Europe focusing on the impact of the first COVID-19 wave on mental health, health behavior, and well-being were restricted to adolescents and children of school age^25–28^.

The main aim of this study is therefore to analyze changes in preschoolers´ social-emotional developmental risks by comparing child-specific data assessed in 2019 before the onset of the COVID-19 pandemic with data assessed in 2020 after the first COVID-19 wave. In addition, data from previous survey waves (2017/2018 and 2018/2019, respectively) were examined to contextualize the changes in preschoolers´ social-emotional developmental risks.

## Methods

### Instrument

Developmental risks were assessed using the revised “Dortmund Developmental Screening for Preschools (Dortmunder Entwicklungsscreening für den Kindergarten, DESK 3–6 R)”. The DESK 3–6 R is an age-specific, standardized, reliable and valid instrument to monitor the developmental risks of 3- to 6-year-old preschool children in the domains of motor, linguistic, cognitive and social development^29–31^.

The DESK 3–6 R is designed in three age versions (one for 3-year-olds, one for 4-year-olds and one for 5-to-6-year-olds) with respective competence domains (see Table 1). It is taken into account that competencies differentiate and develop with each age group.

**Table 1:** DESK 3–6 R competence domains covered according to age group.

3-year-old children	4-year-old children	5–6-year-old children
Fine motor skills	Fine motor skills	Fine motor skills
Gross motor skills	Gross motor skills	Gross motor skills
Social behavior	Social behavior	Social competence
		Social interaction
Cognition and language	Language and communication	Language and communication
		Basic competence written language
	Cognition	Attention and concentration
		Basic competence mathematics

The DESK 3–6 R is completed by the pedagogical staff. There are different task formats applied in the DESK 3–6 R, such as observational tasks, group play tasks and individual tasks. It is advisable that the preschool teacher who knows the child well assesses the DESK 3–6 R. Scores are calculated from the respective tasks, which are transformed to "stanine" (standard nine) values per domain using norm tables, ranging from 1–9 points. The norm tables for the 3–4-year-olds are available at half-yearly age intervals, so that these additionally take age differences within the group into account (age-adjusted). A stanine value of 1 represents a conspicuous result (corresponding to the 1st–4th percentile of the norm sample) and a stanine value of 2 represents an inconclusive result (i.e., 5th–11th percentile). A conspicuous result implies that an expert (e.g., a pediatrician) should be consulted to further examine the child for a developmental risk. If the result is inconclusive, the DESK 3–6 R should be repeated at a later time point. Furthermore, stanine values of 3–9 (i.e., 12th–100th percentile) indicate normal development and are referred to as "no findings" in the following.

The domain social behavior is included in DESK 3–6 R for 3-year-olds and 4-year-olds and consists of nine observation tasks in both age versions, which differ between the age versions (see Table 2). The response options are often/very often, sometimes, and rarely/never. Each task that was met with often/very often corresponds to one screening point, whereas the other response options correspond to zero screening points. A maximum of 9 screening points can be achieved. Regarding the domain social behavior, the DESK 3–6 R has an inter-rater reliability between 69.2% (DESK 3–6 R for 3-year-olds) and 88.2% (DESK 3–6 R for 4-year-olds) and is reliable with a Cronbach’s alpha of α = 0.91 (DESK 3–6 R for 3-year-olds) and α = 0.77 (DESK 3–6 R for 4-year-olds), respectively. Furthermore, the DESK 3–6 has proven valid as a screening instrument^32^. Results in the DESK domain “social behavior” are strongly associated with subscales of the Strengths and Difficulties Questionnaire (SDQ)^33^, e.g. in 3-year-olds with the SDQ-subscale “emotional symptoms” (*r* = − 0.41; *p* < 0.01) and in 4-year-olds with the SDQ-subscale “hyperactivity-impulsivity” (*r* = − 0.59; *p* < 0.01).

**Table 2:** Translated observational tasks for the domain social behavior for 3-year-olds and 4-year-olds.

No	Tasks for 3-year-olds	Tasks for 4-year-olds
1	Turns to reference person in the preschool	Plays constructively and builds up something without immediately destroying it again
2	Speaks of him- or herself in the first-person	Puts away the things after playing with them, if necessary after being asked to do so
3	Can differentiate between boys and girls	Follows the rules of age-appropriate games (board games, card games)
4	Judges the behavior of other children	Reacts adequately to emotional expressions of other children
5	Plays together with two children	Takes an active role in role games
6	Puts away the things after playing with them, if necessary after being asked to do so	Has a temporary (over a few weeks) friendship with another child
7	Knows different places and names them correctly	Considers wishes of other children
8	Says his or her name and knows where he or she lives	Has the confidence to approach other children on his or her own
9	Develops play themes with animal characters or dolls	Wait for his or her turn

### Study design

In this prospective dynamic cohort study, the data were collected as part of the project “Evaluation of the targeted individual promotion in preschools in Mecklenburg-Western Pomerania (GIF M-V)". The project was initiated in 2011 as part of the evaluation of the federal state law for children’s day-care and preschools in Mecklenburg-Western Pomerania (Kindertagesförderungsgesetz—KiföG M-V) and is funded by the Ministry for Social Affairs, Integration and Equality. The primary aim of this project is to identify children with developmental risks at an early stage so that they can receive targeted, tailored, individualized and child-centered support conducted by the pedagogic staff of their preschool, based on reliable information from a scientifically recognized instrument, namely the DESK 3–6 R. To this end, an annual developmental screening is carried out in preschools in economically deprived areas. In general, low socioeconomic status is indicative of social injustice negatively affecting children's school enrollment and academic performance^34^. Therefore, the preschool setting offers an impactful opportunity to target a large number of children and promote children's competencies, thus supporting children’s educational opportunities as they enter school.

The study was approved by the ethics committee of the University Medicine Greifswald (BB109/11). All procedures performed in this study were in accordance with the ethical standards of the institutional and/or national research committee and with the 1964 Helsinki declaration and its later amendments or comparable ethical standards. Informed consent was obtained from all individual participants included in the study.

### Study region

Mecklenburg-Western Pomerania is the north-easternmost federal state in Germany. The unemployment rate with 7.8% is higher than in most other German states (as of 2020, national average: 5.9%)^35^. As such, Mecklenburg-Western Pomerania has the third highest unemployment rate in Germany in 2020^35^. One in five residents in Mecklenburg-Vorpommern was at risk of poverty in 2019 (19.4%, national average: 15.9%)^36^. The proportion of children attending preschool among 3- to 6-year-olds is 95.6% (2020), the second-highest attendance rate in this age group in all of Germany^37^. Participation in preschool is voluntary in Germany; compulsory school attendance begins in the school year when children have turned 6.

With regard to the first COVID-19 wave, Mecklenburg-Western Pomerania was the first German state to introduce restricted regular preschool operation and had one of the highest preschool return rates^2^. As of August 01, 2020, a further opening step has been taken with the introduction of regular operation under pandemic conditions^38^. Thereby, open or partially open childcare programs were permitted, but only in separate, consistent subareas with up to 100 children, whereby the same children were assigned to one pedagogical staff. If possible, the groups should be assigned to fixed rooms.

### Implementation of the project

Each year, the Ministry for Social Affairs, Integration and Equality provides the evaluation team a list of participating DESK-preschools. Those DESK-preschools were selected according to an above-average proportion of parents in a given region, whose parental fees were subsidized by the youth welfare offices. In 2020, N = 152 DESK-preschools were eligible for support based on the federal state law, which represents a proportion of 14% of all preschools in Mecklenburg-Western Pomerania^39^. The preschools participate over a period of at least three years. The preschools can use the annual financial support from the ministry for pedagogical support of the children (preferably for additional staff hours^40^).

The annual implementation of the DESK 3–6 R is required by law for the participating preschools. The parents are asked to give their written informed consent for the data to be forwarded to the project team. This is voluntary. For this project, standardized privacy statements were developed in accordance with the GDPR (General Data Protection Regulation) requirements, as well as a parent information sheet. The consent to forward the data can be revoked at any time with effect for the future. From the time of revocation, the data will no longer be processed. There are no disadvantages in case of non-participation. The consent forms remain in the preschools.

Pedagogical staff at each participating preschool receive training upon enrollment in the project. Subsequently, they annually conduct the DESK 3–6 R from beginning of May until the end of November among all 3–6-year-old children (referred to as one survey wave).

The pedagogical promotion of social-emotional competencies in the participating DESK-preschools in general, but also especially for children with developmental risks is complex. The contents of the intervention in the domain of social behavior have a designated focus on the promotion of social competencies and use standardized programs and/or cooperate with external specialists for this purpose. The program content focuses on strengthening self-confidence, interacting with peers through games, and reflecting and communicating emotions.

### Data management and data protection

The pseudonymization and longitudinal matching of DESK 3–6 R data for each child is carried out before the actual data evaluation in the Independent Trusted Third Party of the University Medicine Greifswald using the ID Management solution E-PIX (Enterprise Identifier Cross Referencing)^41^. E-PIX enables unique participant management and efficient aggregation of research data^42,43^. For child-related evaluations, the child-related data of the subsequent survey years must be reliably linked to each other for this purpose. Therefore, pseudonyms (Master Patient Index (MPI-ID)) are generated that do not change over time, including surname, name, birth date, gender, and preschool ID. This procedure allows, for example, to clearly assign DESK 3–6 R data from one survey year to DESK 3–6 R data from the following survey year and to compare the results from both years on a child-specific level. After linkage, data is provided for analysis in a pseudonymized format. Thus, it is not possible for the project staff to identify the respective child. All identifying information is stored in the Trusted Third Party of the University Medicine Greifswald.

### Data analysis and statistical methods

For the data analysis longitudinal DESK 3–6 R data from 3-year-olds at survey wave 3 (DESK-R-SW3, conducted in 2019) and 4-year-olds at survey wave 4 (DESK-R-SW4, conducted in 2020) were used, each during the period from 1st of August to 30th of November, since regular preschool operation started on August 01, 2020, in Mecklenburg-Western Pomerania concomitantly with the end of the first COVID-19 wave. To quantify the selection bias, we compared prevalence rates of females between cross-sectional data from DESK-SW3 and DESK-SW4 with longitudinal data from females who participated in both SWs. The prevalence ratio to assess sampling bias (PR-SB) was calculated as the difference between the prevalence of females in the longitudinal data set divided by the prevalence of females based on the cross-sectional data. When the prevalences in both groups were equal, the PR-SB was equal to 1. A PR-SB of > 1 indicated overrepresentation (positive bias), and a PR-SB of < 1 indicated underrepresentation (negative bias)^44^. Missing values were excluded from the analysis. The stanine values of the domain social behavior were transformed into a binary variable, whereby stanine values of 1–2 were grouped into the developmental risk/inconclusive finding category and stanine values of 3–9 were categorized as no finding (i.e., normal development).

For descriptive data analysis, absolute and relative frequencies are reported for nominal variables, and the median and 25th and 75th percentiles are reported for continuous variables. For longitudinal univariate analysis McNemar (for categorical variables) test was applied. A *p*-value of *p* < 0.05 was considered statistically significant.

For the calculation of the prevalence rate ratio (PRR) the formula PRR = [a/(a + b)]/[c/(c + d)] was applied, where a = children with developmental risks/inconclusive findings in DESK-R-SW4, b = children without developmental risks in DESK-R-SW4, c = children with developmental risks/inconclusive findings in DESK-R-SW3, and d = children without developmental risks in DESK-R-SW3. Thereby, a PRR < 1 indicates that the proportion of children with “developmental risk/inconclusive finding” decreased from DESK-R-SW3 to DESK-R-SW4 and a PRR > 1 indicates that the proportion increased. A PRR = 1 indicates no change between the two survey waves. The PRR is reported with its 95% confidence interval.

Additionally, we calculated the ratio of the rate of improvements (developmental risk/ inconclusive finding at DESK-R-SW3, normal development at DESK-R-SW4) divided by the rate of deteriorations (normal development at DESK-R-SW3, developmental risk/inconclusive finding in DESK-R-SW4). Thereby, the number of children with improvement was divided by the number of all children with a developmental risk/inconclusive finding at DESK-R-SW3 and compared to children with deterioration divided by all children with normal development at DESK-R-SW3^45^. A ratio > 1 indicates that the rate of improvements is higher than the rate of deteriorations, whereas a ratio < 1 indicates that the rate of improvement is lower than the rate of deteriorations.

In addition, as supplementary analyses, data from 3-year-old children at survey wave 1 (DESK-R-SW1, conducted in 2017) were longitudinally compared with data from 4-year-old children at survey wave 2 (DESK-R-SW2, conducted in 2018), and data from 3-year-olds at survey wave 2 (DESK-R-SW2) were compared with data from 4-year-olds at survey wave 3 (DESK-R-SW3). Here, the same methodology as described above was applied. A Forest plot was generated to visualize the results (PRR) of the different longitudinal analyses.

IBM SPSS Statistics (Version 28, IBM, Armonk, USA) was applied for statistical analysis, and STATA (Version 14.2, StataCorp, College Station, USA) was used to calculate the PRR. The stanine values were calculated using the SAS statistical software package (Version 9, SAS Institute Inc., Cary, USA). The Forest plot was generated with R (version 4.0.4) using the R package ‘metafor’.

## Results

### Developmental risks in the domain social behavior before and after first COVID-19 wave

**Figure 1:**
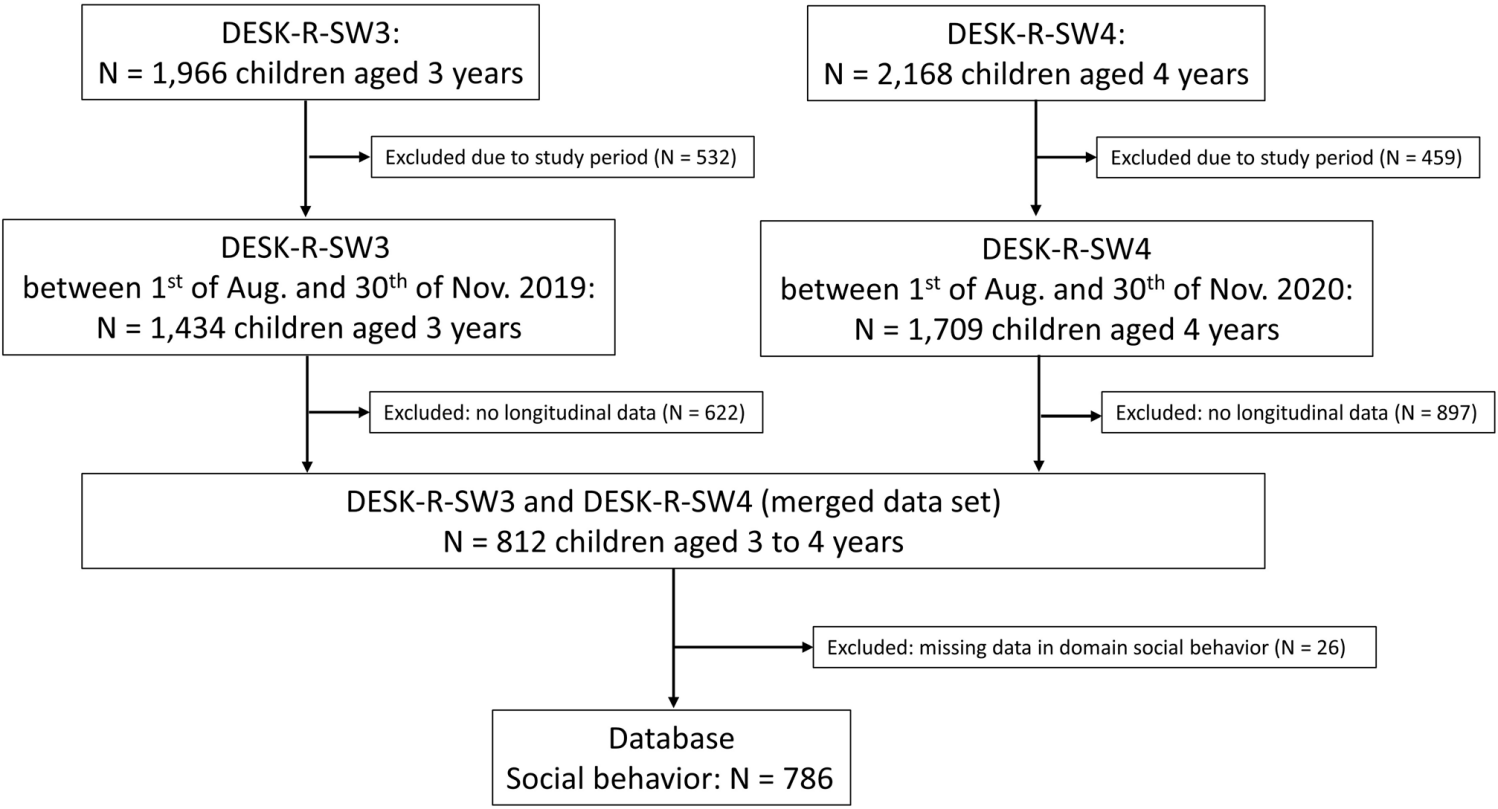
Consort diagram illustrating the data base for longitudinal data analysis of age-adjusted DESK scores in the domain social behavior.

In DESK-R-SW3 and DESK-R-SW4, *N* = 152 preschools participated in the project. In DESK-R-SW3, *N* = 1434 3-year-old children participated in DESK during the period from 1st of August to 30th of November 2019, whereas in DESK-R-SW4 (Aug-Nov 2020), *N* = 1709 4-year-olds participated. Longitudinally assessed data at DESK-R-SW3 and DESK-R-SW4 for the domain social behavior were available for *N* = 786 children (see Fig. 1). With bias estimates regarding female gender of 0.99 (DESK-SW3) and 0.98 (DESK-SW4), we found no indications of sampling bias due to sampling of cases with longitudinal data from all participants (see Supplementary Table S1).

**Figure 2:**
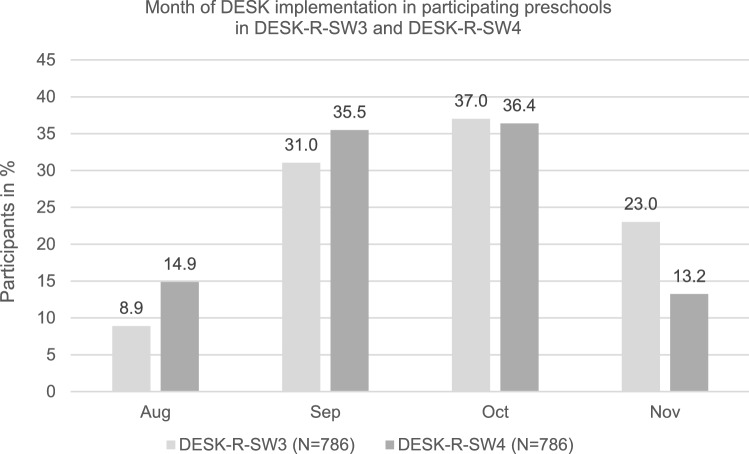
Month of cross-sectional DESK assessment in participating preschools in DESK-R-SW3 (conducted in 2019) for 3-year-olds and DESK-R-SW4 (conducted in 2020) for 4-year-olds.

Consequently, at the time of DESK-R-SW3, children were 3 years old and at the time of DESK-R-SW4, children were 4 years old. Figure 2 shows the monthly distribution of DESK completion for the two survey waves (for the included children in this study only).

In DESK-R-SW3 (before the onset of the COVID-19 pandemic) the proportion of children with developmental risks/inconclusive findings was 18.2%, whereas in DESK-R-SW4 (after the onset of the COVID-19 pandemic) the proportion of children with developmental risks/inconclusive findings was 12.4% (see Table 3). Accordingly, a decrease in the prevalence rate of developmental risks/inconclusive findings was observed when comparing pre-pandemic to after the first COVID-19 wave. The prevalence rate ratio is 0.68 (95% CI [0.54, 0.87], *p* = 0.001).

**Table 3:** Categorized changes of results in the DESK domain “Social behavior” from DESK-R survey wave 3 (DESK-R-SW3; conducted in 2019) to DESK-R survey wave 4 (DESK-R-SW4; conducted in 2020) (N = 786).

Social behavior	DESK-R-SW4 (after the first COVID-19 wave)			PRR	95% CI	*p-*value	Ratio of the rate of improvements^1^ divided by the rate of deteriorations^2^
	No finding	Developmental risk/inconclusive finding	Total				
	n (%)	n (%)	n (%)				
DESK-R-SW3 (pre-pandemic)							
No finding	600 (76.3)	43 (5.5)	643 (81.8)	0.68	[0.54–0.87]	0.001	9.31
Developmental risk/inconclusive finding	89 (11.3)	54 (6.9)	143 (18.2)				
Total	689 (87.6)	97 (12.4)	786 (100)				

*CI* confidence interval, *PRR* prevalence rate ratio, *DESK-R-SW3* DESK-R survey wave 3, *DESK-R-SW4* DESK-R survey wave 4.

^1^Improvements: risk at DESK-R-SW3, no risk at DESK-R-SW4.

^2^Deteriorations: no risk at DESK-R-SW3, risk in DESK-R-SW4.

Furthermore, 76.3% of all children showed no findings in both survey waves, while 6.9% of the children consistently showed developmental risks/inconclusive findings. 11.3% of the children improved (i.e., children with developmental risks/inconclusive findings in DESK-R-SW3 who had no findings in DESK-R-SW4), whereas 5.5% of the children deteriorated (no findings in DESK-R-SW3 but developmental risks/inconclusive findings in DESK-R-SW4). The ratio of the improvement rate (number of children with improvement from DESK-SW3 to DESK-SW4 divided by the number of all children with a developmental risk/inconclusive finding at DESK-R-SW3: 89/143) divided by the deterioration rate (children with deterioration from DESK-SW3 to DESK-SW4 divided by all children with normal development at DESK-R-3: 43/643) resulted in 9.31 (see Table 3), indicating that the proportion of children who improved was 9.31 times higher than the proportion of children who deteriorated.

### Developmental risks in the domain social behavior in previous survey waves

**Figure 3:**
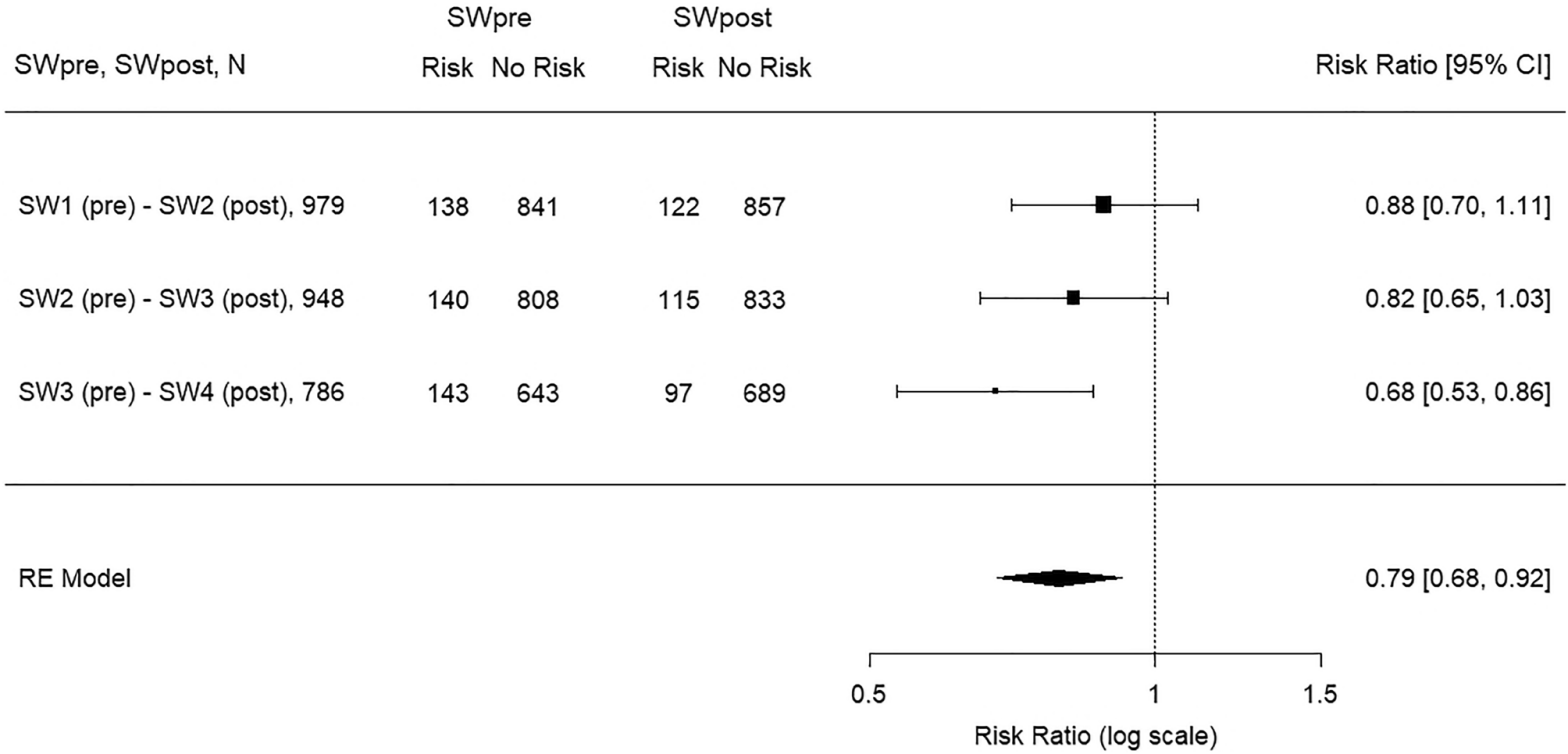
Forest Plot visualizing the prevalence rate ratios (Risk Ratios) of the three longitudinal analyses: 2017 to 2018 (DESK-R-SW1 to DESK-R-SW2, N = 979), 2018 to 2019 (DESK-R-SW2 to DESK-R-SW3, N = 948) and 2019 to 2020 (DESK-R-SW3 to DESK-R-SW4, N = 786); *SW* survey wave, *SWpre* first survey wave of the longitudinal comparison, *SWpost* following survey wave one year after SWpre of the longitudinal comparison; *Risk* number of children with developmental risk/inconclusive finding; *No Risk* number of children with no finding; *CI* confidence interval;

To place the results in the context of the children's individualized targeted promotion as part of this project, two additional longitudinal analyses were conducted using data from previous assessments comparing 2017 to 2018 (DESK-R-SW-1 to DESK-R-SW-2, N = 979) and 2018 to 2019 (DESK-R-SW-2 to DESK-R-SW-3, N = 948) (see Supplementary Tables S2 and S3). The proportion of 3- to 4-year-old-children with developmental risk/inconclusive finding decreased in both longitudinal comparisons with a PRR = 0.88 (*p* = 0.287) for DESK-R-SW-1 to DESK-R-SW-2 and a PRR = 0.82 (*p* = 0.092) for DESK-R-SW-2 to DESK-R-SW-3. The results were additionally visualized with a Forest plot (see Fig. 3). Interestingly, the proportion of children with a persistent developmental risk/inconclusive finding increased gradually over the three longitudinal comparisons (DESK-R-SW-1 to DESK-R-SW-2: 5.0%; DESK-R-SW-2 to DESK-R-SW-3: 5.5%; DESK-R-SW-3 to DESK-R-SW-4: 6.9%), whereby the proportion of children with deterioration gradually decreased (DESK-R-SW-1 to DESK-R-SW-2: 7.5%; DESK-R-SW-2 to DESK-R-SW-3: 6.6%; DESK-R-SW-3 to DESK-R-SW-4: 5.5%). The rate of improvement was relatively constant at 9.1–9.3% in the first two longitudinal comparisons and then increased to 11.3% from DESK-R-SW-3 to DESK-R-SW-4. Over the three longitudinal analysis, the ratio of the rate of improvement divided by the rate of deterioration increased steadily from 7.43 to 9.31, indicating that over the years gradually more children improved than deteriorated. However, one can see that the proportion of 3-year-olds with developmental risks/inconclusive findings (18.2%) at DESK-R-SW-3 was considerably higher than in the previous comparisons (14.1–14.8%).

## Discussion

The development of social behavior in children in the context of the pandemic is influenced by various factors, such as cultural differences between countries, differences in the pandemic course and socioeconomic status of the families, which are, taken all together, difficult to account for in one study. Thus, it is not surprising that previous studies examining differences in children's social behavior during the initial COVID-19 lockdown reached different conclusions. Complicating matters further, the study designs of most previous longitudinal studies differed, sometimes substantially; some examined rather short-term periods of one month, others had small sample sizes, and some studies conducted the baseline assessments during the lockdown^20–24^. To date, there is a lack of clear evidence of the impact of the COVID-19 pandemic on the development of social behavior. The present study examined changes in developmental risk in social behavior among more than 700 preschool children after the first COVID-19 wave using a standardized and validated instrument in a longitudinal comparison from 2019 (before the onset of the COVID-19 pandemic) to 2020 (after the first COVID-19 wave) in relation to results from assessments conducted in previous years.

In general, the development of social-emotional competencies is of paramount importance, as social-emotional functioning in early childhood was found to be a predictor of school readiness and academic achievement through, e.g., self-control, positive attitudes, motivation and social interactions with peers^46^. Social-emotional functioning, moreover, can be considered a basic competency contributing to mental health. Generally, mental health or well-being should be conceptualized not only as the absence of psychopathological symptoms, but also as the presence of competencies, such as social-emotional functioning, that enable individuals to withstand adversity and work toward positive outcomes^47^. Previous studies found that social-emotional problems in preschool were associated with an increased incidence of mental health problems in adolescence, such as anxiety and depression, further highlighting the importance of fostering social-emotional competencies as early as at preschool age^48–50^. Particularly in the context of the COVID-19 pandemic, one might assume that the lack of promotion of social-emotional competencies by pedagogical staff in the preschools had negative effects. In the present study, however, we found no evidence of an increase in social-emotional developmental risks among 3- to 4-year-olds in preschools after the first COVID-19 wave.

Surprisingly, in our study the prevalence of developmental risks decreased from 2019 before the pandemic to 2020 after the first COVID-19 wave. Compared to the longitudinal analyses from the previous survey waves, it should be emphasized that across all comparisons, the proportion of children with improvement was greater than the proportion of children with deterioration (PRR < 1), and since the scales are age-adjusted, this can for the most part be explained by the targeted individual promotion of children with developmental risks. With regard to the first-time significant change in developmental risks in the comparison from before the pandemic to after the first COVID-19 wave, it should be noted that all three longitudinal analyses each examined changes in the domain social behavior among 3- to 4-year-old children and, accordingly, different children are considered in all three comparisons. Thus, differences in the socioeconomic status of families, child promotion, or secular trends across years cannot be ruled out.

The key finding of our study, we would like to highlight, is that we could not detect any deterioration in the prevalence rate ratio after the first COVID-19 wave compared to previous survey waves, whereby several aspects must be considered:

With regard to the COVID-19 pandemic, only short-term effects can be described, since only 4.5 months passed from the start of the nationwide lockdown in mid-March to the first DESK assessment in early August. Mecklenburg-Western Pomerania was also the first German state to allow limited regular care and had one of the highest preschool return rates, so that from the beginning of May every third child was allowed to attend preschool and by the end of May three quarters were already able to attend preschool again^2^. Apart from Saxony, which had a preschool attendance rate of 78% of the children by end of May, all other German federal states ranged between 22 and 47%^2^. However, child care programs were still partially restricted after August 2020, so preschools with a number of more than 100 children could not continue open or partially open child care concepts as they used to before the pandemic. The pedagogical staff was urged to stay with the same children, if possible, and to place children in the same cohorts in fixed rooms so that COVID-19 outbreaks could be avoided. Regarding the development of social behavior, this may have made it easier for children to approach other children in the preschool, strengthening cohesion and creating a more familiar environment.

Due to the more favorable child–adult ratio during the lockdown, closer supervision was provided. Family cohesion may have increased, which may have created a more emotionally stabilizing home environment^21^. On the other hand, one could assume that the COVID-19-induced lockdown may have increased demands on family resources and sociodemographic risks^24^. In general, with regard to the social-emotional development it was found that higher levels of family resources and more sensitive parenting predicted lower levels of aggression, whereas higher sociodemographic risk and less sensitive, less involved parenting, assessed from infancy through third grade, predicted higher and more stable aggression^51^. Studies conducted so far in relation to the pandemic have shown a decreased quality of life of children, an increased stress experience of parents and a lockdown-related increase in mental health problems^52–55^. Furthermore, Sun et al. showed that parental distress significantly predicted both externalizing and internalizing child behavior problems in primary school aged children^56^. Since these studies did not include 3–4-year-old children, however, no clear conclusions can be drawn.

Two studies from the United Kingdom conducted by the same authors with a comparable design found no differences in emotional and behavioral problems in 2–5-year-old children over a one-month period starting during the first lockdown, whereas primary school aged children (4–10 years old) showed an increase in child emotional and behavioral difficulties^22,23^. For secondary school aged children (11–16 years old), emotional difficulties decreased and behavioral difficulties did not change. Therefore, it can be assumed that the lockdown affected the social-emotional development of children in different age groups to different degrees^57^. Accordingly, our findings support results from previous studies that found no deterioration in social behavior among preschool children during the first COVID-19 wave^21,22^.

So far studies in toddlers and preschoolers found, in terms of daily activities, that during early stages of the pandemic, time spent in physical activity decreased, recreational screen time, and sleep duration increased, and sleep quality declined^12,58^. However, Kurz et al. could not find substantial differences in sleep quality, physical activity, and time spent with books in 6–7-year-old children in South Germany during the first COVID-19 wave^59^. One study of 2–4-year-olds estimated that every additional hour per day that children watched TV or digital media is associated with a higher mean SDQ score for conduct problems^60^. Another study in primary school children found that playing and learning activities were linked to lower behavioral problems, active leisure was positively associated with physical well-being, and socialization and family activities were linked to better social well-being^61^.

Furthermore, the study included preschools from economically disadvantaged regions as part of the project. Data from previous studies have shown that economically disadvantaged children of mentally burdened parents are particularly affected by the negative consequences of the lockdown, for example due to cramped housing or lack of parental support and encouragement^52–54,62^. Furthermore, studies found that a lower socioeconomic status was associated with higher media use and less outdoor activity^61,63^.

However, one limitation of this study is that the influence of the household on the change in developmental risks could not be considered, as these were not assessed. This includes social living conditions, families’ socioeconomic status but also the influence of siblings or the childcare situation during the lockdown. A major limitation of the DESK 3–6 R is that not all domains of competence are measured across all age groups, so that the domain social behavior is only determined for 3–4-year-old children. Social behavior is further differentiated in the 5–6-year-olds into social competence and social interaction, so for consistency of the outcome measure we have restricted this analysis to children of 3–4 years. Furthermore, it should be noted that the categorization of the stanine values into two groups (developmental risk vs. no developmental risk) results from the fact that the DESK 3–6 R is rather designed to sensitively identify which children are at risk for developmental risks and need targeted, individualized support. The calculated stanine values are therefore not “performance points”, but primarily indicate whether support is needed or not. Moreover, our study examined only short-term effects of the pandemic on developmental risks in the domain of social behavior from the onset of the pandemic until the end of November 2020.

One major strength of this study is that longitudinal data from a prospective cohort study from over 700 3–4-year-old children in the domain social behavior were assessed using a standardized, validated instrument contextualized with data from previous survey waves. To date, limited data of the impact of the COVID-19 pandemic on developmental risks in this domain are available for this age group. One further major strength of this study is that the instrument was administered by trained pedagogical staff who knew the children over a longer period of time and were therefore able to assess them well. The completion by parents bears the risk that the children are assessed more positively and, in addition, the perception might change during the lockdown due to the increased child-parent time or parental stress.

In conclusion, despite the first COVID-19 wave, no negative effects on developmental risks in the domain of social behavior were observed among 3- to 4-year-old preschool children in economically deprived regions in northeastern Germany. On the contrary, despite preschool closures due to the COVID-19 pandemic, decreases in developmental risks were observed in the longitudinal comparison, although besides the individual, targeted promotion of children with developmental risks provided by preschools in the context of this project, secular trends cannot be completely ruled out. Further studies are needed to examine long-term effects of the pandemic on social behavior in preschool children. Previous studies indicated that social-emotional problems in preschool children can negatively impact many areas of later life, particularly mental health, which has deteriorated significantly in children and adolescents in the context of the COVID-19 pandemic. Therefore, it is imperative to effectively promote preschoolers´ social-emotional competencies and to address developmental risks at an early stage, to promote mental health, prevent mental illness, and to enhance educational achievements.

## Supplementary Information

Supplementary Tables.

## Data Availability

The datasets generated and analyzed during the current study are not publicly available after consultation with the data protection commissioner of Mecklenburg-Western Pomerania due to data protection reasons and contractual regulations. For further information please contact Dr. Marco Franze (Email: marco.franze@uni-greifswald.de).

## References

[1] Blum, S. & Dobrotic, I. *Die Kita- und Schulschließungen in der COVID-19-Pandemie* 81–99 (2021).

[2] *Corona-KiTa-Studie**, **Monatsbericht Juli 2020 der Corona-KiTa-Studie* https://www.dji.de/veroeffentlichungen/aktuelles/news/article/774-corona-kita-studie-registrierung-fuer-die-studie-startet.html.

[3] Götzinger F (2020). COVID-19 in children and adolescents in Europe: A multinational, multicentre cohort study. Lancet Child. Adolesc. Health.

[4] Ehrhardt J (2020). Transmission of SARS-CoV-2 in children aged 0 to 19 years in childcare facilities and schools after their reopening in May 2020, Baden-Württemberg, Germany. Euro Surveill..

[5] Bierman KL, Welsh J, Heinrichs BS, Nix RL (2018). Effect of preschool home visiting on school readiness and need for services in elementary school: A randomized clinical trial. JAMA Pediatr..

[6] Nix RL (2016). The randomized controlled trial of Head Start REDI: Sustained effects on developmental trajectories of social-emotional functioning. J. Consult. Clin. Psychol..

[7] Burchinal M (2020). School-entry skills predicting school-age academic and social–emotional trajectories. Early Childh. Res. Q..

[8] Petermann U, Petermann F (2015). Vorschulalter. Kindheit Entwicklung.

[9] Bask M (2015). Externalising and internalising problem behaviour among Swedish adolescent boys and girls. Int. J. Soc. Welf..

[10] Achenbach TM (1978). The child behavior profile: I. Boys aged 6–11. J. Consult. Clin. Psychol..

[11] Achenbach TM, Edelbrock CS (1978). The classification of child psychopathology: A review and analysis of empirical efforts. Psychol. Bull..

[12] Alonso-Martínez AM, Ramírez-Vélez R, García-Alonso Y, Izquierdo M, García-Hermoso A (2021). Physical activity, sedentary behavior, sleep and self-regulation in Spanish preschoolers during the COVID-19 lockdown. Int. J. Environ. Res. Public Health.

[13] Christner N, Essler S, Hazzam A, Paulus M (2021). Children's psychological well-being and problem behavior during the COVID-19 pandemic: An online study during the lockdown period in Germany. PLoS ONE.

[14] Luijten MAJ (2021). The impact of lockdown during the COVID-19 pandemic on mental and social health of children and adolescents. Qual. Life Res..

[15] Loades ME (2020). Rapid systematic review: The impact of social isolation and loneliness on the mental health of children and adolescents in the context of COVID-19. J. Am. Acad. Child. Adolesc. Psychiatry.

[16] Singh S (2020). Impact of COVID-19 and lockdown on mental health of children and adolescents: A narrative review with recommendations. Psychiatry Res..

[17] Racine N (2021). Global prevalence of depressive and anxiety symptoms in children and adolescents during COVID-19: A meta-analysis. JAMA Pediatr..

[18] Pustake M (2022). Have the COVID-19 pandemic and lockdown affected children's mental health in the long term? A repeated cross-sectional study. BMJ Open.

[19] Browne DT (2021). Children’s mental health problems during the initial emergence of COVID-19. Can. Psychol..

[20] Specht IO, Rohde JF, Nielsen AK, Larsen SC, Heitmann BL (2021). Changes in emotional-behavioral functioning among pre-school children following the initial stage Danish COVID-19 lockdown and home confinement. Front. Psychol..

[21] Hagihara H (2022). COVID-19 school and kindergarten closure relates to children's social relationships: A longitudinal study in Japan. Sci. Rep..

[22] Shum, A., Pearcey, S., Dodd, H., & Lawrence, P. *Changes in Pre-school Children’s Emotional and Behavioural Difficulties Through Lockdown in North West England (Report 04). Co-SPYCE study.* https://cospaceoxford.org/findings/emotional-behavioural-difficulties-in-nwe-oct-2020/ (2020).

[23] Pearcey, S., Shum, A., Raw, J., Waite, P., Patalay, P., & Creswell, C. *Changes in Children and Young People’s Emotional and Behavioural Difficulties Through Lockdown (Report 04). Co-SPACE Study.* https://cospaceoxford.org/findings/4th-update/ (2020).

[24] Linnavalli T, Kalland M (2021). Impact of COVID-19 restrictions on the social-emotional wellbeing of preschool children and their families. Educ. Sci..

[25] Viner R (2022). School closures during social lockdown and mental health, health behaviors, and well-being among children and adolescents During the first COVID-19 wave: A systematic review. JAMA Pediatr..

[26] Marchi J, Johansson N, Sarkadi A, Warner G (2021). The impact of the COVID-19 pandemic and societal infection control measures on children and adolescents' mental health: A scoping review. Front. Psychiatry.

[27] Bignardi G (2020). Longitudinal increases in childhood depression symptoms during the COVID-19 lockdown. Arch. Dis. Child..

[28] Achterberg M, Dobbelaar S, Boer OD, Crone EA (2021). Perceived stress as mediator for longitudinal effects of the COVID-19 lockdown on wellbeing of parents and children. Sci. Rep..

[29] Biermann J, Franze M, Hoffmann W (2020). Social developmental delays among 3 to 6 year old children in preschools in German social hotspots: Results of a dynamic prospective cohort study. BMC Pediatr..

[30] Tröster H, Flender J, Reineke D (2011). Prognostische Validität des Dortmunder Entwicklungsscreening für den Kindergarten (DESK 3–6). Diagnostica.

[31] Wolf SM, Tröster H (2017). DESK 3–6 R. Frühe Bildung.

[32] Heinrich Tröster, J. F., Dirk Reineke, Sylvia Mira Wolf. *Dortmunder Entwicklungsscreening für den Kindergarten—Revision*. (Hogrefe, 2016).

[33] Goodman R (1997). The strengths and difficulties questionnaire: A research note. J. Child Psychol. Psychiatry.

[34] Lehti H, Erola J, Karhula A (2019). The heterogeneous effects of parental unemployment on siblings’ educational outcomes. Res. Soc. Stratif. Mobil..

[35] *Arbeitslose und Arbeitslosenquoten - Deutschland, Länder, Kreise und Gemeinden (Zeitreihe Monats- und Jahreszahlen)*, https://statistik.arbeitsagentur.de/SiteGlobals/Forms/Suche/Einzelheftsuche_Formular.html?submit=Suchen&topic_f=gemeinde-arbeitslose-quoten (2021).

[36] *Sozialberichterstattung**: **Armutsgefährdungsquote - gemessen am Bundesmedian und am Landesmedian*, https://www.destatis.de/DE/Themen/Gesellschaft-Umwelt/Soziales/Sozialberichterstattung/Tabellen/liste-armutsgefaehrungsquote-bundeslaender.html;jsessionid=589C7BA8125810EA17D445AC89AAC14D.live712

[37] *Statistisches Jahrbuch Mecklenburg-Vorpommern – Ausgabe 2021, Kinder- & Jugendhilfe**, **Kapitel 7*. https://www.laiv-mv.de/static/LAIV/Abt4.Statistisches%20Amt/Dateien/Publikationen/Statistisches%20Jahrbuch/Aktuell%20nach%20Kapiteln/7%20Kinder-%20und%20Jugendhilfe.pdf

[38] *Corona-KiTa-Studie, 1. Quartalsberichtbericht der Corona-KiTa-Studie (III/2020)*. https://corona-kita-studie.de/monatsberichte-der-corona-kita-studie (2020).

[39] Kathrin Bock-Famulla, A. M., *et al*. *Länderreport Frühkindliche Bildungssysteme 2021*. https://www.laendermonitor.de/fileadmin/files/laendermonitor/laenderprofile_2021/Laenderprofil_MV_2021.pdf (2021).

[40] Franze M (2018). Reducing developmental risks by additional staff hours: Effects of a government program to support day care centers with socially deprived children. Child. Youth Serv. Rev..

[41] Unabhängige Treuhandstelle der Universitätsmedizin Greifswald. Unser Record-Linkage. E-PIX 2019. https://www.ths-greifswald.de/forscher/e-pix/.

[42] Bialke M (2015). MOSAIC: A modular approach to data management in epidemiological studies. Methods Inf. Med..

[43] Bialke M (2015). A workflow-driven approach to integrate generic software modules in a Trusted Third Party. J. Transl. Med..

[44] Nohr EA, Liew Z (2018). How to investigate and adjust for selection bias in cohort studies. Acta Obstet. Gynecol. Scand..

[45] Franze M, Biermann J, Kästner A, Ernst VS, Hoffmann W (2022). Effects of the targeted intervention for five- to six-year-old children affected by attentional and concentration developmental risks: Results of a dynamic prospective cohort study conducted in socially deprived Regions in Germany. Prev. Sci..

[46] Jones DE, Greenberg M, Crowley M (2015). Early social-emotional functioning and public health: The relationship between kindergarten social competence and future wellness. Am. J. Public Health.

[47] Mondi CF, Giovanelli A, Reynolds AJ (2021). Fostering socio-emotional learning through early childhood intervention. Int. J. Child Care Educ. Policy.

[48] Thomson KC (2019). Association of childhood social-emotional functioning profiles at school entry with early-onset mental health conditions. JAMA Netw. Open.

[49] Klein AM (2019). Latent trajectories of internalizing symptoms from preschool to school age: A multi-informant study in a high-risk sample. Dev. Psychopathol..

[50] Meagher SM, Arnold DH, Doctoroff GL, Dobbs J, Fisher PH (2009). Social-emotional problems in early childhood and the development of depressive symptoms in school-age children. Early Educ. Dev..

[51] NICHD Early Child Care Research Network (2004). Trajectories of physical aggression from toddlerhood to middle childhood: Predictors, correlates, and outcomes. Monegr. Soc. Res. Child Dev..

[52] Ravens-Sieberer U (2021). Quality of life and mental health in children and adolescents during the first year of the COVID-19 pandemic: results of a two-wave nationwide population-based study. Eur. Child Adolesc. Psychiatry.

[53] Ravens-Sieberer U (2020). Mental health and quality of life in children and adolescents during the COVID-19 pandemic-results of the Copsy study. Dtsch. Arztebl. Int..

[54] Ravens-Sieberer U (2021). Impact of the COVID-19 pandemic on quality of life and mental health in children and adolescents in Germany. Eur. Child Adolesc. Psychiatry.

[55] Cusinato M (2020). Stress, resilience, and well-being in Italian children and their parents during the COVID-19 pandemic. Int. J. Environ. Res. Public Health.

[56] Sun J (2022). Child behavior problems during COVID-19: Associations with parent distress and child social-emotional skills. J. Appl. Dev. Psychol..

[57] Schmidt SJ, Barblan LP, Lory I, Landolt MA (2021). Age-related effects of the COVID-19 pandemic on mental health of children and adolescents. Eur. J. Psychotraumatol..

[58] Aguilar-Farias N (2020). Sociodemographic predictors of changes in physical activity, screen time, and sleep among toddlers and preschoolers in chile during the COVID-19 pandemic. Int. J. Environ. Res. Public Health.

[59] Kurz D, Braig S, Genuneit J, Rothenbacher D (2022). Lifestyle changes, mental health, and health-related quality of life in children aged 6–7 years before and during the COVID-19 pandemic in South Germany. Child Adolesc. Psychiatry Ment. Health.

[60] Li X (2021). Screen use and mental health symptoms in canadian children and youth during the COVID-19 pandemic. JAMA Netw. Open.

[61] Oliveira VH, Martins PC, Carvalho GS (2022). Children’s daily activities and well-being during the COVID-19 lockdown: Associations with child and family characteristics. Curr. Psychol..

[62] Amerio A (2020). COVID-19 Lockdown: Housing built environment's effects on mental health. Int. J. Environ. Res. Public Health.

[63] Poulain T (2021). Loss of childcare and classroom teaching during the Covid-19-related lockdown in spring 2020: A longitudinal study on consequences on leisure behavior and schoolwork at home. PLoS ONE.

