# Supplementary material for: Changes in social behavioral developmental risks in preschool children after the first COVID-19 wave: a prospective longitudinal cohort study
Source: Sci Rep. 2023 Apr 6;13:5615. doi: 10.1038/s41598-023-32877-x (PMC10078017; doi:10.1038/s41598-023-32877-x)
Supplement: Supplementary file 1 — Supplementary Tables. [file 41598_2023_32877_MOESM1_ESM.pdf]

Changes in social behavioral developmental risks in preschool children after the first COVID-19 wave: a prospective longitudinal cohort study

Kästner A[1*], Ernst VS[1], Hoffmann W[1], Franze M[1]

[1] Institute for Community Medicine, Section Epidemiology of Health Care and Community Health, University Medicine Greifswald, Germany

Supplementary Table S1 – Results of sampling bias analysis

	Cross-sectional data SW3 (N=1,966)	Longitudinal data SW3-SW4 (N=786)	PR-SB[1]
Female Gender, in %	49.1%	48.5%	0.99
	**Cross-sectional data SW4 (N=2,168)**	**Longitudinal data SW3-SW4 (N=786)**	**PR-SB[1]**
Female Gender, in %	49.5%	48.5%	0.98

Note: [1]PR-SB = prevalence ratio to assess sampling bias

Supplementary Table S2 - Categorized changes of results in the DESK domain “Social behavior” from DESK-R survey wave 1 (DESK-R-SW1; conducted in 2017) to DESK-R survey wave 2 (DESK-R-SW2; conducted in 2018) (N = 979)

Social behavior		DESK-R-SW2			PRR	95% CI	*p*-value	Ratio of the rate of improvements[1] divided by the rate of deteriorations[2]
		No finding	Developmental risk / inconclusive finding	Total				
		n (%)	n (%)	n (%)				
**DESK-R-SW1**	No finding	768 (78.4)	73 (7.5)	841 (85.9)	0.88	[0.70-1.11]	0.287	**7.43**
	Developmental risk / inconclusive finding	89 (9.1)	49 (5.0)	138 (14.1)				
	Total	857 (87.5)	122 (12.5)	979 (100)				

Note: CI = confidence interval; PRR = Prevalence rate ratio; DESK-R-SW1 = DESK-R survey wave 1; DESK-R-SW2 = DESK-R survey wave 2; [1] Improvements: risk at DESK-R-SW1, no risk at DESK-R-SW2; [2]Deteriorations: no risk at DESK-R-SW1, risk in DESK-R-SW2

Supplementary Table S3 – Categorized changes of results in the DESK domain "Social behavior" from DESK-R survey wave 2 (DESK-R-SW2; conducted in 2018) to DESK-R survey wave 3 (DESK-R-SW3; conducted in 2019) (N = 948)

Social behavior		DESK-R-SW3			PRR	95% CI	*p*-value	Ratio of the rate of improvements[1] divided by the rate of deteriorations[2]
		No finding	Developmental risk / inconclusive finding	Total				
		n (%)	n (%)	n (%)				
**DESK-R-SW2**	No finding	745 (78.6)	63 (6.6)	808 (85.2)	0.82	[0.65-1.03]	0.092	**8.06**
	Developmental risk / inconclusive finding	88 (9.3)	52 (5.5)	140 (14.8)				
	Total	833 (87.9)	115 (12.1)	948 (100)				

Note: CI = confidence interval; PRR = Prevalence rate ratio; DESK-R-SW2 = DESK-R survey wave 2; DESK-R-SW3 = DESK-R survey wave 3; [1] Improvements: risk at DESK-R-SW2, no risk at DESK-R-SW3; [2]Deteriorations: no risk at DESK-R-SW2, risk in DESK-R-SW3
